# Exploring the Microbial Landscape: Gut Dysbiosis and Therapeutic Strategies in Pancreatitis—A Narrative Review

**DOI:** 10.3390/biomedicines12030645

**Published:** 2024-03-14

**Authors:** Vasile Valeriu Lupu, Roxana Mihaela Bratu, Laura Mihaela Trandafir, Laura Bozomitu, Gabriela Paduraru, Nicoleta Gimiga, Gabriela Ghiga, Lorenza Forna, Ileana Ioniuc, Florin Dumitru Petrariu, Bogdan Puha, Ancuta Lupu

**Affiliations:** Faculty of Medicine, “Grigore T. Popa” University of Medicine and Pharmacy, 700115 Iasi, Romania; valeriulupu@yahoo.com (V.V.L.); trandafirlaura@yahoo.com (L.M.T.); laura.bozomitu@gmail.com (L.B.); chiti_nico@yahoo.com (N.G.); ghiga.gabriela@yahoo.com (G.G.); lorenza.donea@yahoo.ro (L.F.); ileanaioniuc@yahoo.com (I.I.); florin.petrariu@umfiasi.ro (F.D.P.); puhab@yahoo.com (B.P.); anca_ign@yahoo.com (A.L.)

**Keywords:** gut microbiota, pancreatitis, dysbiosis, gut–pancreas axis, microbiota-based treatment

## Abstract

The gut microbiota is emerging as an important contributor to the homeostasis of the human body through its involvement in nutrition and metabolism, protection against pathogens, and the development and modulation of the immune system. It has therefore become an important research topic in recent decades. Although the association between intestinal dysbiosis and numerous digestive pathologies has been thoroughly researched, its involvement in pancreatic diseases constitutes a novelty in the specialized literature. In recent years, growing evidence has pointed to the critical involvement of the pancreas in regulating the intestinal microbiota, as well as the impact of the intestinal microbiota on pancreatic physiology, which implies the existence of a bidirectional connection known as the “gut–pancreas axis”. It is theorized that any change at either of these levels triggers a response in the other component, hence leading to the evolution of pancreatitis. However, there are not enough data to determine whether gut dysbiosis is an underlying cause or a result of pancreatitis; therefore, more research is needed in this area. The purpose of this narrative review is to highlight the role of gut dysbiosis in the pathogenesis of acute and chronic pancreatitis, its evolution, and the prospect of employing the microbiota as a therapeutic intervention for pancreatitis.

## 1. Introduction

The digestive system has the largest density of microorganisms in the entire human body, populated by over 10^14^ microorganisms such as bacteria, fungi, viruses, and protozoa, all of which comprise the gastrointestinal microbiota [1]. Molecular studies have shown that the gastrointestinal microbiome comprises over 1000 bacterial species, containing 100 times more genes than the human genome. Over 99% of the bacteria are represented by five primary phyla: *Firmicutes* (synonym *Bacilliota*), *Bacteroidetes* (synonym *Bacteroidota*), *Proteobacteria* (synonym *Pseudomonadota*), *Verrucomicrobiota*, and *Actinobacteria* (synonym *Actinomycetota*) [2,3].

The microbiota, also known as “the forgotten organ”, has long been disregarded in medical practice. Through its involvement in nutrition and metabolism, protection against pathogens, and immune system modulation [4], the gut microbiota has been the object of increasing interest, becoming one of the main topics of study in the last few decades. Although, beginning at birth [5,6], the gut microbiota composition varies substantially among healthy individuals depending on age, sex, comorbidities, genetic predisposition, and dietary factors, maintaining a status of “eubiosis” is possible due to a balance between commensal flora and the immune system, with growing evidence that proves the gut microbiota to be a critical determinant of homeostasis in the human body [7]. Alterations in the composition of the microbiome, with the occurrence of dysbiosis, contribute to the initiation and evolution of a diverse range of pathologies, both gastrointestinal and metabolic [7,8], such as inflammatory bowel diseases [3], celiac disease [9], irritable bowel disease [10], obesity and diabetes mellitus [11], and various cancers with gastrointestinal localization. In addition, extra-intestinal pathologies, like allergies [12], heart diseases [2], asthma [13], chronic kidney disease [14,15], and systemic lupus erythematosus [16] have occurred, but the involvement of the microbiome in diseases of organs such as the liver or the pancreas represents a novelty in the specialized literature.

Pancreatic inflammatory pathologies, represented mainly by acute and chronic pancreatitis, represent a challenge in clinical practice due to their fulminant or chronic evolution, high mortality rates, and their associated complications; the early estimation of disease severity as well as the use of antibiotic therapy are intensely debated topics. Multiple studies have demonstrated the connection between the pancreas and the microbiome; through the interaction of the immune system, pro-inflammatory state, and dysbiosis, any change at any of those levels induces a response in the other component, which suggests the existence of a bidirectional connection between the pancreas and the intestine, known as the “gut–pancreas axis” [17,18].

Current evidence indicates significant shifts in the gut microbiota following the onset of pancreatitis, albeit with a limited number of studies suggesting microbial variations across different etiologies and severities. Although our knowledge of the composition of the gut microbiota and its alterations in pancreatitis has increased in recent years, a significant gap persists in comprehending the role of the microbiome in disease severity and progression and its viability as a therapeutic target by subsequently integrating these insights into clinical practice.

In light of its sustained significance as a focal point of research in the last few years, this narrative review summarizes recent advances in the study of the gut microbiota and its implications in pancreatitis and analyzes the potential role of the gut microbiota in the etiopathogenesis of acute and chronic pancreatitis, focusing on human studies and covering the clinical and physiological mechanisms involved. Additionally, we discuss the possibility of using the microbiota as a potential therapeutic intervention for pancreatitis.

## 2. Materials and Methods

The scientific literature was browsed using the Scopus, PubMed, and Embase databases by searching the following keywords: “acute pancreatitis”, “chronic pancreatitis”, “gut microbiota”, and “dysbiosis” in various combinations. The search was limited to open-access articles published in English within the last 10 years. Inclusion criteria encompassed studies primarily concentrating on delineating comprehensive alterations in the gut microbiota among human subjects with acute and chronic pancreatitis. Articles that were vague and did not focus on specific aspects of the subject were excluded from the review process, but they were utilized for general informational purposes.

## 3. Gut Microbiota Profile in Pancreatitis

Pancreatitis has a global distribution with an increasing incidence, with reported values of approximately 15–73 cases per 100,000 people for acute pancreatitis [19], while chronic pancreatitis has an estimated incidence of 4.4–14 cases per 100,000 people [20], with variations in the incidence depending on the geographical location, gender, or age, as well as on the etiology [21]. Considered in the past to be a sterile organ, the pancreas was found to have a pancreatic microbiome through the translocation of bacteria from the duodenum to the pancreatic tissue via the pancreatic ductal system or the mesenteric lymphatic system and the venous drainage [1].

It is well known that the pancreas influences the microbiota directly through the secretion of antimicrobial peptides, and indirectly through the involvement of pancreatic enzymes in the digestion process, thus playing an important role in its composition [22]. The antimicrobial peptides (AMPs) are secreted in healthy conditions by the pancreatic acinar cells through the pancreatic juice and contribute significantly in modulating the intestinal microbiota, being indispensable for innate immunity [22]. These peptides are believed to restrict the access of commensal and pathogenic bacteria to intestinal epithelia [4]. Their insufficient secretion has been associated with excessive growth in intestinal bacteria and the emergence of a pro-inflammatory status, while the intestinal microbiota influences the secretion of AMPs by decreasing the levels of short-chain fatty acids (SCFAs), metabolites produced by the gut microbiota (for example, butyrate, propionate, acetate, isovalerate, valerate, and hexanoate), which have an anti-inflammatory role. In experimental studies, it was shown that by binding with G-protein-coupled receptor-43 (GPR43), butyrate activates anti-inflammatory cytokine production, such as tumor necrosis factor-beta (TGF-β) and interleukin-10 (IL-10), as well as upregulates the FOXP3, a member of the forkhead transcription factor family of Treg cells. The knockout of the receptor GPR43 increased susceptibility to dextran-sodium-sulfate-induced colitis in mice by increasing chemotaxis of neutrophils and inflammatory gene expression [4]. SCFAs are considered to be vital components in maintaining the integrity of the intestinal barrier [23]. Studies have shown that SCFAs can promote the intestinal epithelial thigh junction protein synthesis, inhibit intestinal permeability, and even enhance the intestinal mucosal immune barrier function [24].

### 3.1. Acute Pancreatitis

Acute pancreatitis (AP) is defined as an acute inflammatory process caused by inadequate activation of trypsinogen and other proteolytic enzymes, resulting in pancreatic acinar injury and a local inflammatory response [17,25]. This is a frequent cause of hospital emergency admission with an elevated socio-economic burden. The most common causes of acute pancreatitis are obstruction of the biliary tract (40%) and alcohol consumption (30%), followed by metabolic (hyperlipidemia) and post-traumatic causes [17,26]. The evolution of pancreatitis is often limited, with a moderate dysfunction as is found in interstitial pancreatitis, but it is complicated by the appearance of pancreatic necrosis and persistent organ dysfunction in approximately 35% of cases [27]. According to the Atlanta Classification revised in 2012, acute pancreatitis is classified as moderate AP (MAP), moderately severe AP (MSAP), and severe AP (SAP) [28].

**Figure 1:**
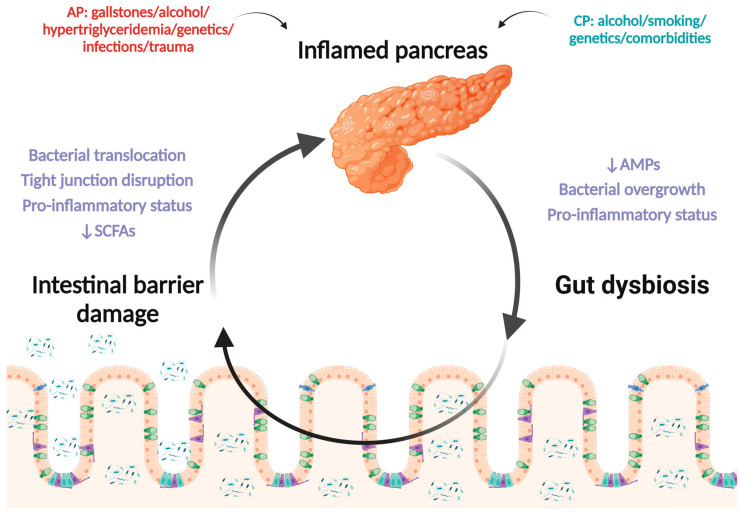
Bidirectional gut–pancreas axis alteration in pancreatitis. (Created with BioRender.com). Abbreviations: AMPs—antimicrobial peptides, SCFAs—short-chain fatty acids, AP—acute pancreatitis, CP—chronic pancreatitis.

Regardless of the etiology, the pathogenesis of pancreatitis involves activation, leading to abnormal secretion of pancreatic enzymes. This is accompanied by a local inflammatory response through the release of pro-inflammatory cytokines, for example, tumor necrosis factor-alpha (TNF-α) and interleukin-1β (IL-1β), which further amplifies the inflammatory response, leading to the development of a systemic inflammatory syndrome [29,30]. By affecting the microcirculation at the intestinal level, followed by a decrease in tissue perfusion, the integrity of the intestinal barrier is altered [29]. Numerous studies have demonstrated that intestinal permeability increases shortly after the onset of pancreatitis, a process followed by the translocation of bacteria to other locations (Figure 1) [31,32].

Wu and colleagues reported in a meta-analysis that approximately 59% of patients with acute pancreatitis experienced a dysfunction of the intestinal barrier [33], while Li et al. reported that 68.8% of patients had blood-circulating bacterial DNA specific to the microbiota found in pancreatitis [34]; these two processes were shown to have an important prognostic value [35]. Bacterial translocation is responsible for the occurrence of secondary infectious complications both locally and systemically, with an important impact on mortality. In a meta-analysis, Werge et al. observed that the mortality of patients with infected pancreatic necrosis and organ dysfunction was twice as high compared to that of patients with sterile pancreatic necrosis and organ dysfunction [36].

#### 3.1.1. Gut Microbiota at the Onset of Acute Pancreatitis

Selected studies (Table 1) reported changes in the intestinal microbiota since the onset of acute pancreatitis, describing a decrease in bacterial diversity, with an increase at the phylum level in potentially pathogenic bacteria such as *Bacteroidetes* and *Proteobacteria* compared to healthy controls [32,37,38,39,40]. At the family level, *Enterococcaceae*, *Enterobacteriaceae*, and *Bacteroidaceae* increased, while at the genus level, *Bacteroides*, *Enterococcus*, and *Escherichia–Shigella* were more abundant in patients with AP [32,40,41]. A decrease at the phylum level in *Firmicutes* and *Actinobacteria* was reported [38], while at the genus level, a decrease in *Bifidobacterium* and *Blautia* was observed [32,40,41]; at the family level, a decrease in *Ruminococcaceae* in patients with AP versus healthy controls was observed [32].

Multiple studies have shown that these bacterial alterations are positively correlated with disease severity [37,40,41,42]. Hu et al. reported that the abundance of *Enterococcaceae* and *Enterobacteriaceae* increased with disease progression [32], while Yu et al. and Zhu et al. observed that the dominant species varied according to the degree of disease severity, with *Bacteroides* being abundant in the mild form, and *Escherichia–Shigella* in the moderate–severe form, while *Enterococcus* was more predominant in the severe form [37,45]. *Proteobacteria* was also found to be significantly increased with disease severity, with research demonstrating *Proteobacteria* overgrowth in patients with AP, particularly SAP [37,40,45].

Hu and colleagues found that *Escherichia–Shigella* had moderate to strong positive correlations with intensive care unit (ICU) admission and hospitalization, acute necrotic accumulation, walled-off necrosis, and shock, and had a strong positive correlation with infected pancreatic necrosis, disorders of consciousness, and death [43]. *Enterococcus* was positively associated with sepsis, infection, liver damage, ICU admission and hospitalization, organ failure, and shock in patients with hypertriglyceridemia-associated acute pancreatitis compared to non-hypertriglyceridemia-associated acute pancreatitis [31,43].

In another study, which compared the microbiota composition between healthy controls, patients with acute pancreatitis and acute respiratory distress syndrome (ARDS), and patients with acute pancreatitis without ARDS, the gut microbiome had the characteristics found in ARDS-AP patients even before they were diagnosed [32]. These results suggest the possibility of using a microbiota analysis test as a biomarker for early recognition of severe pancreatitis and for establishing the prognosis. However, further studies are needed to confirm the specificity and sensitivity of this method [32,41].

A very limited selection of studies compared the different etiologies of the alterations in the gut microbiota in acute pancreatitis. Hu et al. reported significant differences in gut microbiota composition and function in patients with hypertriglyceridemia (HTGAP)-associated acute pancreatitis compared to those with non-HTGAP. These microbiota changes were related to the poor prognosis of HTGAP [43], which may suggest that the alterations in gut microbiota patterns are influenced not only by the pancreatitis itself, but also by its causative factor [43,44]. Although the specific mechanisms by which hypertriglyceridemia exacerbates the progression of AP remain unclear, excessive production of free fatty acids is hypothesized to lead to oxidative stress, vascular endothelial injury, pancreatic necrosis, and systemic inflammatory response [43,46,47].

Changes in the gut microbiota have also been correlated with metabolite alterations and immune dysfunction; multiple studies have shown that an overgrowth of potentially pathogenic bacteria affects the immune system by its involvement in the downregulation of Tregs cells, Th2 cells, and B cells, and through promoting the production of pro-inflammatory factors, such as IL-1β, IL-6, and TNF-α [32], which are believed to play an important role in the systemic complications of AP. Tan et al. observed that the plasma endotoxin concentrations and serum levels of TNF-α, IL-6, IL-10, and IL-1 in patients with severe acute pancreatitis were significantly higher compared to the control group and patients with moderate–acute pancreatitis (MAP). Furthermore, it was outlined that the serum levels of TNF-α and IL-1 were significantly higher in the MAP group compared to the healthy controls. In addition, the IL-6 serum concentration was significantly increased in patients with severe acute pancreatitis with associated gut microbiota alterations compared to patients with severe pancreatitis without changes in the gut microbiota [41].

Toll-like receptors (TLRs) are key players in innate immune response, believed to play an important role in AP, as they are thought to be the major receptors for recognizing bacteria. Qi-Xiang et al. reported that the deletion of TLR4 in the gut epithelium was associated with exacerbated intestinal and pancreatic injury during AP, which could be attributed to gut dysbiosis and Paneth cell dysfunction. They also observed that *L. reuteri* activated the Paneth cells and promoted epithelial proliferation, which might suggest that *Lactobacillus* supplementation might be a possible therapeutic principle against AP [48,49].

#### 3.1.2. Gut Microbiota on AP Complications

AP complications are associated with high mortality rates and are believed to be caused by circulating bacteria, which can cause local infections within necrotic areas of the pancreas or can aggravate the systemic inflammatory response with the occurrence of organ failure. Selected studies followed and presented the alterations in the composition of the gut microbiota in two of the most challenging complications in clinical practice: necrotizing pancreatitis and acute pancreatitis-related acute respiratory distress syndrome (AP-ARDS).

Necrotizing acute pancreatitis (NP) is reported to occur in 20–30% of patients with severe pancreatitis and leads to an unfavorable prognosis, with a reported mortality rate of 15% or higher for those with infected NP (30–39%) [31]. It was previously believed that the microbiota composition of infected pancreatic necrosis was dominated by Gram-negative flora originating in the lower gastrointestinal tract, such as *Enterobacteriaceae* [32]. Recent studies have shown that the widespread use of prophylactic antibiotics from the early stages has shifted the dominant flora to *Staphylococcus*, *Enterococcus*, and *Candida* [32]. Zou and colleagues observed that *Enterococcus* was more abundant in patients with necrotizing pancreatitis, while bacteria with probiotic properties, such as *Bacteroides*, were less abundant [31]. They also reported that an altered microbiota was predictive of a worse outcome for NP patients, while *Finegoldia magna* and *Enterococcus faecium* emerged as promising biomarkers for NP and infected NP [31]. However, the interplay between the pathophysiology of NP and these microorganisms must be further investigated. Yang et al. and Yanagibashi et al. demonstrated in their studies that *Bacteroides* plays an essential role in modulating the IgA production in the digestive tract and in restricting the pathogenic bacteria and endotoxins from invading gut epithelial cells [50,51].

ARDS is defined as a type of acute inflammatory lung injury, with an estimated rate of mortality of 48% due to a delayed or missed diagnosis in approximately two-thirds of patients [52]. As previously mentioned, the gut microbiota has been proven to have the characteristics of ARDS in AP patients even before diagnosis. Some changes were thought to be related to the onset and development of AP-ARDS, such as the enrichment of *Enterobacteriaceae* and *Escherichia–Shigella*, and the reduction of *Bifidobacterium* was associated with AP-ARDS [32]. In previous studies, gut-associated bacteria, especially *Bacteroidetes* and *Enterobacteriaceae*, were found to be abundant in the lower respiratory tract of ARDS patients and were correlated with serum IL-6 levels and in-hospital mortality in patients with ARDS [53,54].

### 3.2. Chronic Pancreatitis

Chronic pancreatitis (CP) is a fibro-inflammatory condition of the pancreas, characterized in evolution by irreversible, progressive changes, which cause variable degrees of exocrine and endocrine dysfunctions [20]. It is a multifactorial pathology, and the most frequent causes of chronic pancreatitis include toxic and metabolic (alcohol, hyperlipidemia, drugs), genetic, autoimmune, obstructive, and idiopathic elements [20]. Chronic pancreatitis places a significant burden on patients, as well as on health systems, with a negative influence on patients’ quality of life and life expectancy [55]. CP patients have an increased risk of pancreatic cancer and impaired mental health [56]. In addition to the signs of exocrine pancreatic insufficiency manifested by varying degrees of malnutrition, these patients describe non-specific symptoms such as abdominal distention, accompanied by a potentially disabling diffuse abdominal pain syndrome, flatulence, and symptoms suggestive of small intestinal bacterial overgrowth [55]. The collection of studies included in this review (Table 2) reports a decrease in bacterial diversity in patients with CP compared to healthy controls [55,56] and an increase in susceptibility to the occurrence of bacterial overgrowth syndrome in the small intestine (SIBO) [55,57]. The etiology of CP-related SIBO is unknown; suggested mechanisms include a combination of altered anatomy, altered motility, and decreased secretion of pancreatic trypsin, which can affect the activation of defensin, therefore inhibiting pancreatic antibacterial activity [58,59]. In addition, the occurrence of SIBO has been linked to gastrointestinal surgery [60] and the consumption of alcohol and narcotics [59]. Studies also reported an association between diabetes mellitus and SIBO, which is attributed to small bowel enteropathy and neuropathy, resulting in intestinal stasis, which predisposes to bacterial proliferation [61]. Bacterial overgrowth in the small intestine is a recurrent finding in chronic pancreatitis and has been reported to play an important role in the symptom severity, degree of malnutrition, and morbidity rate. In a systematic review by Capurso et al., the prevalence of SIBO was estimated to be approximately 36% of patients with CP, the imbalance of the colonic microbiota being a factor in aggravating symptoms [62].

At the genus level, *Enterococcus* and *Bacteroides* were the most abundant, while the greatest reduction in CP cases was found for *Faecalibacterium* and *Provotella* [55]. Studies have also reported increased levels of pathogenic bacteria such as *Citrobacter*, *Enterobacter*, *Enterobacteriaceae*, *Escherichia–Shigella*, *Klebsiella*, *Pseudomonas*, *Staphylococcus*, and *Streptococcus*. Frost and colleagues observed a 2.8-fold and 5.0-fold increase in the mean and median levels in patients with chronic pancreatitis compared to healthy controls [55]. They also found that smoking was a contributing factor to the levels of facultative pathogens in CP patients, while other phenotypic factors such as age, sex, BMI, diabetes mellitus, or pancreatic elastase levels did not show statistical correlations [55].

*Enterococcus* is a genus with quantitatively increased pathogenic potential among patients with chronic pancreatitis. Experimental studies demonstrated that its presence in increased abundance predisposes patients to the systemic dissemination of the bacteria, conferring an increased risk of systemic complications [67].

This observation could explain why *Enterococcus* was found in high levels in the aspirate of pancreatic pseudocysts [68] and even in the alveolar aspirate of patients with acute pancreatitis and ARDS [32]. It was also observed by Frost et al. that the bacteriostatic effect of metronidazole in combination with the Gram-negative activity of Ceftriaxone appeared to influence *Enterococcus* overgrowth in three of four patients, which is assumed to substantially increase the risk of subsequent *Enterococcus*-caused septicemia [55].

Selected studies unanimously report that modulating T-cell responses, IL-10, and IL-8 markedly decreased the level of *Faecalibacterium prausnitzii*, one of the most abundant bacteria in the commensal microbiota, with anti-inflammatory potential [56,65]. It is postulated that through its involvement in the production of acetate, it is essential for the proliferation and growth of colonic epithelial cells, it can improve the barrier function of the intestinal mucosa, and its reduction affects the integrity of the intestinal mucosa, directly proportional to the duration and severity of the disease and of the associated complications [65].

*Bifidobacterium*, well known for its probiotic properties, was also found in low levels. It is hypothesized that by restoring *Bifidobacterium* levels, we can improve not only the immune response of patients with severe acute pancreatitis but also their nutritional status, indicating that its use could be a central therapeutic target in the management of these pathologies [69].

Wang and colleagues examined four other bacterial genera, namely *Subdoligranulum*, *Phascolarctobacterium*, *Eubacterium*, and *Collinsella*, and their low abundance was associated with glycemic control, inflammatory processes, and also with various gastrointestinal neoplasms [56]. However, it is important to note that an increase in the level of *Subdoligranulum* species was observed after vitamin D administration in patients with Crohn’s disease, suggesting the possibility of using vitamin D supplements in the management of chronic pancreatitis [70].

*Phascolarctobacterium* represents a bacterial genus with pro-inflammatory potential, whose presence in an increased quantity is correlated with the appearance and development of inflammation in metabolic diseases, as well as in psychological ones [70,71]. It was observed that patients with nephrolithiasis had higher levels of *Phascolarctobacterium* compared to healthy controls, and its presence positively correlated with high values of serum trace elements such as potassium, sodium, calcium, and chlorine [72].

*Eubacterium* is a butyrate-producing bacterial genus known for its probiotic role; therefore, it is a key element in maintaining the integrity of the intestinal barrier. Low levels of *Eubacterium* have been reported in Crohn’s disease [73], and increased levels have been observed in primary biliary cholangitis [74].

Wang et al. studied the correlation between the presence of a certain type of microbiota and genetic changes in children diagnosed with chronic pancreatitis, and the results indicated that *Ruminococcaceae*, *Veillonella*, *Butyricicoccus*, and *Phascolarctobacterium* are associated with different functional gene mutations that are related to chronic pancreatitis in children, thus confirming that specific functional gene mutations can affect the composition of the gut microbiota [56].

Jandhyala et al. observed an increased level of bacterial lipopolysaccharide (LPS) and plasma endotoxins in patients with chronic pancreatitis compared to healthy volunteers, despite low levels of *Bacteroides*, a Gram-negative bacterial genus, an important source of LPS. Therefore, the authors postulated that the presence of increased levels of LPS could be determined by the existence of unclassified bacterial species. The potential of LPS to induce inflammation in pancreatic β-cells by activating Toll-like receptors and nuclear factor κ-B, followed by cell destruction and the appearance of diabetes, a complication frequently encountered in patients with chronic pancreatitis, is well known [65].

When it comes to comparing the etiology of gut microbiota alteration in chronic pancreatitis, there is a lack of research on the role of each causal factor in gut dysbiosis. Ciocan et al. reported significant differences in the composition of the gut microbiome in patients with severe alcoholic hepatitis (sAH), chronic alcoholic pancreatitis (CAP), and alcoholic controls (AC) [63]. The authors observed that the microbiota of the patients with chronic alcoholic pancreatitis had a different composition and structure compared to the AC and sAH groups. It was also observed that the relative abundances of potential pathogenic bacteria, such as *Enterococcus*, *Klebsiella*, and *Pseudomonas*, which are associated with systemic inflammations and infection, were greater in CAP patients than in AC patients [63].

It is widely accepted that the gut microbiome alterations in patients with chronic pancreatitis are merely the outcome rather than the cause of the disease. These alterations are thought to be mediated by chronic inflammatory pathways or by changes in the composition of the intestinal chyme, leading to changes in the availability of substrates for microbial metabolism [55].

**Figure 2:**
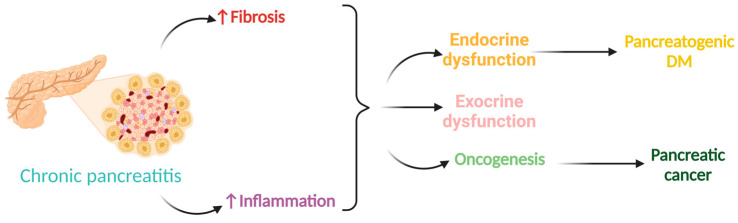
CP involvement in pancreatic cancer and diabetes mellitus (DM).

Although the changes observed in the intestinal microbiome are not correlated with exocrine pancreatic dysfunction, as shown in some studies [55,65], we cannot exclude the possibility that the gut microbiota directly affects the progression and severity of CP. Recent studies on malignant pancreatic diseases underline the input of the gut microbiota in the oncogenesis process and disease evolution by its involvement in the tumor microenvironment [75,76]. Therefore, it is believed that by similar mechanisms, CP can promote pancreas inflammation and subsequent fibrosis (Figure 2) [55], leading to progressive and irreversible focal, segmental, or diffuse lesions within both exocrine and endocrine pancreatic tissue. CP is one of the main risk factors for the occurrence of pancreatic cancer, followed by alcohol and tobacco consumption [77].

In recent years, multiple studies have reported an association between gut dysbiosis and pancreatic cancer, with increased levels of *Bacteroides*, and decreased levels of *Firmicutes*, while at higher taxonomic levels, *Veillonellaceae* was predominant and *Clostridiaceae* was decreased. It was observed that smoking cessation results in an increase in *Firmicutes* and a decrease in *Bacteroides* and *Proteobacteria*, thus highlighting the importance of tobacco abstinence in a lifestyle intervention for cancer patients [78].

Several studies have demonstrated the existence of an intratumor microbiome by DNA analysis indicating an abundance of *Fusobacterium* in patients with pancreatic ductal adenocarcinoma, as well as other types of gastrointestinal cancers [79]. A diverse intratumor microbiome enriched with *Pseudoxanthomonas*, *Streptomyces*, *Bacillus clausii*, and *Saccharopolyspora* was correlated with favorable survival in multiple cohorts [80].

Through lesions within endocrine pancreatic tissue, diabetes mellitus secondary to CP is an important complication that is diagnosed in 30–40% of adults, with an exceptionally high cumulative incidence during CP follow-up [81]. Apart from the micro and cardiovascular complications of diabetes, this type of diabetes, also known as pancreatogenic diabetes or type 3c diabetes mellitus, is associated with additional comorbidities such as maldigestion and malnutrition, having greater morbidity and mortality than type 1 or type 2 diabetes [82].

## 4. Microbiota-Based Treatment as a Possible Therapeutic Target in Pancreatitis

Therapeutic principles used in pancreatitis are restricted to symptomatic treatment, which includes the supplementation of pancreatic enzymes and the management of associated complications. Given its role in the homeostasis of the gut microbiota and the immune system, microbiota-based treatment may be effective as a therapeutic target in pancreatitis by promoting products such as probiotics, prebiotics, or even fecal microbiota transplantation [83].

### 4.1. Probiotics

According to the 2013 International Scientific Association for Probiotics and Prebiotics (ISAPP) consensus, probiotics are defined as “live microorganisms that, when administered in adequate amounts, confer a health benefit” [84]. The most abundant bacteria with a probiotic role in the human gut are *Lactobacillus* and *Bifidobacterium*. Probiotics can influence the gut microbiota directly by inhibiting pathogens or indirectly by inducing the production of mucus and AMPs, enhancing the tight junctions and attenuating epithelial cell apoptosis [45]. As previously mentioned, probiotics can modulate the immune system, so they are believed to play an important role in treating pancreatitis.

Oláh et al. conducted the first clinical trial on using probiotics (live *Lactobacillus plantarum* versus heat-killed *Lactobacillus plantarum*) for the treatment of pancreatitis. The researchers reported that live *Lactobacillus plantarum* was effective in reducing infected pancreatic necrosis, sepsis, and the number of surgical interventions [85]. Subsequently, Oláh et al. compared the use of four species of lactic acid bacteria (*Lactobacillus plantarum*, *Lacticaseibacillus paracasei*, *Pediococcus pentosaceus*, and *Leuconostoc mesenteroides*) associated with four prebiotic fibers versus the use of prebiotics alone. They observed that the symbiotic therapy significantly decreased the cumulative incidence of systemic inflammatory response and multiorgan failure and increased the recovery rate of patients [86]. However, Besselink et al. reported that the use of a multi-strain probiotic mixture (*Lactobacillus casei*, *Lactobacillus acidophilus*, *Lactococcus lactis*, *Lactobacillus salivarius*, *Bifidobacterium lactis*, and *Bifidobacterium bifidum*) not only failed to decrease the risk of infectious complications but was also correlated with a higher mortality risk by increasing the occurrence of organ failure [35]. Because the study included more patients with organ failure than any previous trial, the same authors reported in a later analysis that the presence of organ failure modulates the effect of probiotic administration, being linked to an increase in bacterial translocation and enterocyte damage [87]. In contrast to this finding, it was also reported that probiotic prophylaxis in patients without organ failure did not influence enterocyte damage but reduced bacterial translocation [87].

In a recent systematic review, Gao et al. investigated the impact of probiotics on organ failure, mortality, and the length of hospital stay, identifying a statistically significant reduction in the duration of hospital stay (*p* value of 0.010) but not in mortality or the risk of organ failure [88], results that align with the findings published by Malik et al. [89]. In summary, the use of probiotics is still controversial, and extensive studies are needed.

### 4.2. Prebiotics

Prebiotics were defined by the 2016 ISAPP consensus as a “substrate that is selectively utilized by host microorganisms conferring a health benefit” and are represented by conjugated linoleic acids, polyunsaturated fatty acids, and oligosaccharides, for example, fructo-oligosaccharides, galacto-oligosaccharides, inulin, mannan-oligosaccharides, human-milk-oligosaccharides, xylo-oligosaccharides, phenolics, and phytochemicals [90].

Mei and colleagues observed that by administrating chitosan oligosaccharides to mice for four weeks prior to induction of severe acute pancreatitis, the extent of the pancreatic injury was reduced by decreasing oxidative stress [91], while Karakan et al. proved that through the use of prebiotic fiber supplementation via nasojejunal enteral nutrition in patients with severe acute pancreatitis, the acute phase response and the overall complications were reduced [92], therefore proving the use of prebiotics to be beneficial.

### 4.3. Fecal Microbiota Transplantation

Fecal microbiota transplantation (FMT) is a procedure in which stool from a healthy donor is placed into the intestine of another patient [45,83], and it is believed to help restore gut dysbiosis. It has been successfully used as a treatment for recurrent *Clostridioides difficile* infections, and it has proven to be effective in ulcerative colitis, irritable bowel syndrome, and hepatic encephalopathy [83]. Hu et al. demonstrated in a case report that FMT has the potential to be a viable therapeutic strategy for patients with moderately severe acute pancreatitis [93]. However, Zhu et al. reported that practicing FMT in both antibiotic-treated mice and germ-free mice with aggravated AP led to significant pancreatic injury [40]; therefore, the efficacy of FMT in pancreatitis remains to be determined.

## 5. Antibiotic Therapy in Pancreatitis: Friend or Foe?

Antibiotic therapy is often used prophylactically in clinical practice to inhibit the growth of pathogenic bacteria in complicated acute pancreatitis, thus reducing the infectious complications of SAP [94]. Current guidelines [95,96,97,98] recommend against the use of routine antibiotic prophylaxis in severe AP without accompanying cholangitis or infected necrosis, recent studies even showing the lack of positive impact on reducing AP mortality, surgical interventions, or the incidence of infected pancreatic necrosis [99,100], but the use of antibiotic therapy as prophylaxis was associated with a decrease in the incidence of nonpancreatic infections [99] and a reduction in the length of hospital admission [100].

Although not recommended, the clinical use of prophylactic antibiotics has become widespread, and studies report an antibiotic usage rate of 41–88% [101]. This global practice is correlated with significant risks, including increased prevalence of multi-drug-resistant bacteria and increased incidence of fungal infection [30].

The American Gastroenterological Association Institute recommends the use of carbapenems, metronidazole, and quinolones, known to penetrate pancreatic tissue, in infected necrosis, which could delay the need for surgical interventions and reduce mortality and morbidity rates [96]. They also recommend that the optimal duration of antibiotic therapy and the decision to continue/discontinue antibiotic therapy for complicated AP should be determined based on the severity of infection, clinical response, and radiologic findings [96]. Although the use of metronidazole and quinolones, known to have excellent coverage of anaerobic microorganisms, may significantly disturb microbiota diversity, the use of probiotics can prevent the intensification of dysbiosis severity [102].

Certain empiric antibiotics used in hospitalized patients could alter the dominant microbiota by reducing the abundance of Gram-negative organisms in the gastrointestinal tract, leading to the overgrowth of Gram-positive bacteria such as *Enterococcus*, which, as mentioned before, increases the risk of septicemia [31]. Current guidelines support the use of empiric antibiotics with both aerobic and anaerobic Gram-negative and Gram-positive bacteria coverage [30]. It is important to emphasize that the use of antibiotic therapy in AP should be part of an inclusive care approach that includes early hydration, pain management, and nutritional support, but also assessment of the need for invasive interventions, for example, drainage of infected collections or even necrosectomy, if the circumstances require it [95].

## 6. Future Directions

While significant progress has been made in elucidating the involvement of gut dysbiosis in the onset and progression of acute and chronic pancreatitis, a notable gap persists in understanding the potential utility of the microbiota as a prognostic biomarker and as a therapeutic target and, in the end, effectively integrating these insights into clinical practice. Additionally, there remains the necessity to comprehensively characterize gut microbial alterations according to the varied etiopathogeneses of pancreatitis, thus facilitating a tailored therapeutic approach. To address these knowledge gaps, large-scale, multicenter, randomized controlled trials are essential, particularly focusing on the pediatric population, where empirical studies are currently lacking.

Furthermore, with the increasing significance of genomics, proteomics, and metabolomics, their integration into studies pertaining to pancreatitis may represent the next frontier in elucidating the underlying mechanisms and developing tailored treatments based on individual patient characteristics, aiming for more targeted and effective therapies.

## 7. Conclusions

Pancreatitis represents a challenge in clinical practice due to its unpredictable evolution, associated with significant mortality and morbidity rates. Growing data suggest that pancreatitis is a complex disease triggered by the gut microbiota, the immune system, and other environmental and genetic factors, the bidirectional connection and balance between the gut and pancreas being an important link in the research field.

A low diversity of the gut microbiota, combined with an increased quantity of pathogenic bacteria and a decreased abundance of commensal bacteria, has been linked to the development of acute and chronic pancreatitis and their associated implications. The few studies on this issue have documented that the structure and functions of the gut microbiota vary according to the etiology of pancreatitis. However, there has been insufficient research in this area; further investigation into these discrepancies may provide useful insights concerning the complicated etiology of pancreatitis.

Selected studies have highlighted a positive correlation between bacterial alterations in acute pancreatitis and the severity of the disease. Notably, the gut microbiome exhibits characteristics found in patients diagnosed with ARDS even before the diagnosis is established. Moreover, certain bacterial species have been linked to the occurrence of sepsis, organ failure, and ICU admissions. Alterations in the gut microbiota have also been correlated with changes in metabolite profiles and immune dysfunction, thereby exacerbating pancreatic inflammation.

Current research on gut dysbiosis in chronic pancreatitis indicates a reduction in bacterial diversity, with SIBO emerging as a recurrent finding. This dysbiotic state is frequently associated with symptom severity, malnutrition, and a high morbidity rate. Moreover, the sustained inflammatory state characteristic of CP has been linked to the oncogenesis process and endocrine dysfunctions. CP serves as a significant risk factor predisposing individuals to the development of pancreatic cancer or pancreatogenic diabetes.

Despite the relationship between the gut microbiota and pancreatitis not being fully understood, it has been suggested that the gut microbiota, through the use of probiotics, prebiotics, and FMT, could be used as a therapeutic target to reestablish the intestinal barrier and prevent and mitigate complications in AP patients. However, additional studies are needed to demonstrate the efficacy and safety of these methods, in addition to exploring novel and innovative strategies.

## Figures and Tables

**Table 1:** Summary of included studies on the gut microbiota in acute pancreatitis.

Author	Year	Study Design	Disease	Study Population	Material	Microbiota Alterations
Zou et al. [31]	2022	prospective observational single-center	Acute necrotizing pancreatitis	20 HC, 58 AP (19 NP, 39 non-NP) adults	fecal sample (rectal swab), 16s rRNA sequencing	-Order: ↑*Lactobacillales* ↓*Bifidobacteriales* ↓*Clostridiales*
Hu et al. [32]	2023	prospective observational cohort study single-center	Acute pancreatitis	20 HC, 65 AP adults	fecal sample (rectal swab), 16s rRNA sequencing	-Phylum: ↑*Proteobacteria* ↑*Bacteroidetes* -Family: ↑*Enterobacteriaceae* ↑*Enterococcaceae* ↑*Bacteroidaceae* ↑*Clostridiales incertae sedis XI* ↓* Ruminococcaceae* -Genus: ↑*Escherichia–Shigella* ↑*Enterococcus* ↑*Klebsiella* ↓*Bifidobacterium* ↓*Blautia*
Yu et al. [37]	2020	prospective observational	Acute pancreatitis	20 HC, 20 MAP, 20 MSAP, 20 SAP adults	fecal sample (rectal swab), 16s rRNA sequencing	↑*diversity* -MAP: ↑*Bacteroides* ↑*Finegoldia* ↓*Blautia* -MSAP: ↑*Anaerococcus* ↑*Escherichia-Shigella* ↓*Eubacterium hallii* -SAP: ↑* Enterococcus* ↓*Eubacterium hallii*
Zhang et al. [38]	2018	case–control	Acute pancreatitis	44 HC, 45 AP adults	fecal sample, 16s rRNA sequencing	-Phylum: ↑*Bacteroidetes* ↑*Proteobacteria* ↓*Firmicutes* ↓*Actinobacteria*
Zhao et al. [39]	2023	prospective observational single-center	Acute pancreatitis	16 HC, 16 MAP adults, Mice *	duodenal biopsy, 16s rRNA sequencing	-Phylum: ↑*Proteobacteria* ↑*Firmicutes* -Genus: ↑ *Streptococcus* ↑* Neisseria*
Zhu et al. [40]	2019	prospective	Acute pancreatitis	35 HC, 41 MAP, 59 MSAP, 30 SAP adults	fecal sample, 16s rRNA sequencing serum, cytokines	-Phylum: ↑*Proteobacteria* ↓*Bacteroidetes* -Genus: ↑*Escherichia-Shigella* ↑*Enterococcus* ↓*Protevolla* ↓*Faecalibacterium* ↓*Bifidobacterium*
Tan et al. [41]	2015	prospective multicenter	Acute pancreatitis	32 HC, 32 MAP, 44 SAP	fecal sample, 16s rRNA sequencing plasma, endotoxin and cytokine levels	-Family: ↑*Enterobacteriaceae* -Genus: ↑* Enterococcus* ↓* Bifidobacterium*
Yu et al. [42]	2021	prospective observational single-center	Acute pancreatitis	3 HC, 3 MAP, 3 MSAP, 3 SAP adults	fecal sample (rectal swab), shotgun metagenomic sequencing	-MAP: ↑*Streptococcus* ↓*Anaerostipes hadrus* -MSAP: ↑*Unclassified Bacteria* ↓*Anaerostipes hadrus* -SAP: ↑* Enterococcus* ↓*Blautia*
Hu et al. [43]	2021	prospective observational cohort study single-center	Hypertriglyceridemia-associated acute pancreatitis	30 HTGAP, 30 PA non-HTG adults	fecal sample (rectal swab), 16s rRNA sequencing	-Phylum: ↑*Firmicutes (HTGAP)* ↑*Bacteroidetes* ↑*Proteobacteria* -Family: ↑*Enterococcaceae* ↑*Clostridiales incertae sedis XI (HTG)* ↑*Lachnospiraceae (non-HTG)* ↑*Bacteroidaceae (non-HTG)*
Philips et al. [44]	2019	prospective observational	Acute alcoholic pancreatitis	HC, 29 AH, 7 AAP, 7 ABD adults	fecal sample, 16s rRNA sequencing	-Phylum: ↑*Bacteroidetes* ↑*Proteobacteria* ↓*Firmicutes* ↓*Actinobacteria* -Species: ↑*Enterobacter* ↑*Bacteroides* ↓*Lactobacillus* ↓*Fecalibacterium*

Abbreviations: HC—healthy controls, AP—acute pancreatitis, SAP—severe acute pancreatitis, MAP—moderate acute pancreatitis, MSAP—moderate–severe pancreatitis, HTGAP—hypertriglyceridemia acute pancreatitis, AH—alcoholic hepatitis, AAP—acute alcoholic pancreatitis, ABD—acute biliary disease, NP—necrotizing pancreatitis, rRNA—ribosomal RNA, * beyond the scope of this study.

**Table 2:** Summary of included studies on the gut microbiota in chronic pancreatitis.

Author	Year	Study Design	Disease	Study Population	Material	Microbiota Alterations
Frost et al. [55]	2020	prospective observational	Chronic pancreatitis	102 HC, 51 CP Adults	fecal sample, 16s rRNA sequencing fecal pancreatic elastase	↓diversity -Genus: ↑*Enterococcus* ↑*Bacteroides* ↑*Klebsiella* ↑*Pseudomonas* ↑*Escherichia–Shigella* ↑*Staphylococcus* ↑*Streptococcus* ↓*Faecalibacterium* ↓*Prevotella*
Wang et al. [56]	2020	prospective observational single-center	Chronic pancreatitis	35 HC, 30 CP Children	fecal sample, 16s rRNA sequencing	↓diversity -Genus: ↓*Faecalibacterium* ↓*Bifidobacterium* ↓*Phascolarctobacterium* ↓* Butyricicoccus*
Ciocan et al. [63]	2018	prospective observational two centers	Alcoholic chronic pancreatitis	24 CAP, 13 sAH, 45 AC Adults	fecal sample, 16s rRNA sequencing	↓diversity -Phylum ↑*Proteobacteria* ↓*Bacteroides* ↓*Fusobacteria* -Genus: ↑*Klebsiella* ↑*Enterococcus* ↑*Sphingomonas* ↑*Pseudomonas* ↑*Enterococcus* ↑*Flavobacterium* ↓*Lactobacillus* ↓*Faecalibacterium*
Hamada et al. [64]	2018	prospective observational	Chronic pancreatitis	8 CP, 12 AIP Adults	fecal sample, 16s rRNA sequencing	-Species: ↑*Bacteroides ovatus* ↑*Streptococcus australis* ↑*Streptococcus gordonii* ↑*Clostridium lactatifermentans* ↑*Clostridium lavalense*
Jandhyala et al. [65]	2017	prospective observational	Chronic pancreatitis	10 HC, 16 CP, 14 CP-DM Adults	fecal sample, 16s rRNA sequencing plasma, endotoxin levels, fecal elastase assay	-Phylum: ↓*Bacteroidetes* ↑*Firmicutes* -Species: ↑*unclassified bacteria* ↓*Faecalibacterim prausnitzii* ↓*Ruminococcus bromii*
Zhou et al. [66]	2020	prospective observational single-center	Chronic pancreatitis	69 HC, 71 CP	fecal sample, 16s rRNA sequencing, fecal elastase, ELISA	-Phylum: ↑*Proteobacteria* ↓*Firmicutes* ↓*Actinobacteria* -Genus: ↑*Escherichia–Shigella* ↑*Parabacteroides* ↓*Faecalibacterium* ↓*Subdoligranulum*

Abbreviations: HC—healthy controls, CP—chronic pancreatitis, CAP—chronic alcoholic pancreatitis, sAH—severe alcoholic hepatitis, AC—alcoholic controls, AIP—autoimmune pancreatitis, DM—diabetes mellitus, rRNA—ribosomal RNA.

## Abbreviations

AAP	acute alcoholic pancreatitis
ABD	acute biliary disease
AC	alcoholic controls
AH	alcoholic hepatitis
AIP	autoimmune pancreatitis
AMP	antimicrobial peptide
IL-1β	interleukin 1β
AP	acute pancreatitis
ARDS	acute respiratory distress syndrome
ARDS—AP	acute pancreatitis associated acute respiratory distress syndrome
BMI	body mass index
CAP	chronic alcoholic pancreatitis
CP	chronic pancreatitis
DM	diabetes mellitus
DNA	deoxyribonucleic acid
FMT	fecal microbiota transplantation
FOXP3	forkhead box P3
GPR43	G-protein-coupled receptor-43
HC	healthy controls
HTGAP	hypertriglyceridemia acute pancreatitis
ICU	intensive care unit
IL-1	interleukin 1
IL-8	interleukin 8
IL-6	interleukin 6
IL-10	interleukin 10
ISAPP	International Scientific Association for Probiotics and Prebiotics
LPS	bacterial lipopolysaccharide
MAP	moderate acute pancreatitis
MSAP	moderate–severe pancreatitis
non-HTGAP	non-hypertriglyceridemia acute pancreatitis
non-NP	non-necrotizing pancreatitis
NP	necrotizing pancreatitis
rRNA	ribosomal RNA
SAP	severe acute pancreatitis
SCFAs	short-chain fatty acids
sAH	severe alcoholic hepatitis
SIBO	small intestine bacterial overgrowth
T reg	regulatory T cell
Th2	T helper 2
TLRs	Toll-like receptors
TNF-α	tumor necrosis factor-alpha
TGF-β	tumor necrosis factor-beta
